# Sweet, Fat and Salty: Snacks in Vending Machines in Health and Social Care Institutions in Slovenia

**DOI:** 10.3390/ijerph17197059

**Published:** 2020-09-27

**Authors:** Urška Rozman, Igor Pravst, Urška Pivk Kupirovič, Urška Blaznik, Primož Kocbek, Sonja Šostar Turk

**Affiliations:** 1 Faculty of Health Sciences, University of Maribor, Žitna ulica 15, 2000 Maribor, Slovenia; primoz.kocbek@um.si (P.K.); sonja.sostar@um.si (S.Š.T.); 2 Nutrition Institute, Tržaška cesta 40, 1000 Ljubljana, Slovenia; igor.pravst@nutris.org (I.P.); urska.pivk.kupirovic@nutris.org (U.P.K.); 3 National Institute of Public Health, Trubarjeva 2, 1000 Ljubljana, Slovenia; urska.blaznik@nijz.si

**Keywords:** snacks, vending machines, social care institutions, health care institutions, Slovenia

## Abstract

Vending machines in health and social care facilities are often the only possible choice for a quick snack for workers and visitors, in many cases providing unhealthy dietary choices. Our study aimed to analyse the variety and nutritional quality of foods available in vending machines placed in social and health care institution in Slovenia. The available snacks were quantitatively assessed, using traffic light profiling. The model used for nutrient profiling was that of the Food Standards Australia New Zealand (FSANZ). Vending machines in 188 institutions were surveyed, resulting in 5625 food-items consisting of 267 unique product labels. Sweet products dominate in vending machines offers (about 70%), while nuts and seeds (8.4%), yoghurts (2.1%), fruits (1.4%) and milk (0.3%) are present in a very small proportion or are not available at all. According to FSANZ, 88.5% of all displayed food items in vending machines can be considered as lower nutritional quality or less healthy products. The authors’ future activities will be focused on ensuring wider availability of healthy dietary choices and on including official guidelines in tender conditions for vending machines in health and social care institutions in Slovenia.

## 1. Introduction

Since first introduced in the 19th century, vending machines have become extremely popular means for distributing food products. The main advantage of marketing such foodstuffs, which is being exploited today, is their convenience and time-saving. For time-poor hospital staff and visitors to health and social care institutions, vending machines are often the only choice for a quick snack, which is usually energy-dense and nutrient-poor [1,2,3,4,5,6,7,8].

High intake of sweet, fat and salty products contributes significantly to the aetiology of major chronic diseases, including obesity, cardiovascular diseases, diabetes and some cancers [9,10,11,12,13,14,15,16]. The majority of public health programs worldwide target nutritional recommendations, which include limitations on fat, salt and sugar in dietary intake. Internationally, the WHO tackles this problem with the “Action Plan for the Prevention and Control of Non-Communicable Diseases in the WHO European Region 2016–2025” [17]. In Europe, the “Strategy for Europe on Nutrition, Overweight and Obesity-related health issues” [18] and “European Food and Nutrition Action Plan 2015–2020” [19] have also been implemented. However, levels of specific constituents in foods are sometimes also regulated. For example, in 2018 Slovenia joined certain EU countries, which restricted the content of trans fatty acids (TFA) in food by adopting new rules that regulate maximum permitted levels of TFA in foodstuffs [20].

Foods and beverages sold in vending machines are increasingly being scrutinised, therefore calls for local authorities to develop programs to promote healthier offers in vending machines have been made overseas [21,22,23,24,25,26]. Restricting high-calorie options and promotion of healthier choices can lead to better consumer choices in vending machines [27,28,29,30]. In Slovenia, the National Assembly adopted the “National Program on Nutrition and Health Enhancing Physical Activity 2015–2025” [31] which is coordinated by the Ministry of Health. Proper nutrition, physical activity and healthy dietary choices for patients, staff and visitors in health and social care institutions are emphasised as prerequisites for successful treatment [31]. Health and social care institutions, being a public organisation, can provide unique opportunities to model best practice food-supply policies as part of the government’s leadership to promote healthy eating [32]. The aim of our study was to analyse the variety and nutritional quality of foods available in vending machines placed in social and health care institution in Slovenia. The resulting guidelines for providing healthier choices for hospital workers, patients and visitors could also support future regulatory interventions.

## 2. Materials and Methods

### 2.1. Data Collection

We conducted a cross-sectional survey of all accessible vending machines offering foods in Slovenian health and social care institutions from June to August 2018. A total of 188 locations were identified and visited; 26 were hospitals, 64 health centres and 98 nursing homes. One nursing home did not give access. Data collection based on face-front items, i.e., an item in a slot that was next in line to be sold, in each vending machine [1]. Additionally, in case of five or more empty item slots the survey was repeated for the specific vending machine on another occasion. Non-standard vending machines (e.g., freshly squeezed orange juice or warm beverage vending machines) were excluded in further analysis.

For each vending machine the information about the region, type and name of institution, exact location (e.g., department) and vending machine provider was recorded. Food items consisted of 267 unique product labels and presented 45.8% (*n* = 2579) of the total of 5625 face-front items. According to the Global Food Monitoring Group food categorisation system [33], all identified foods were assigned in one of the 11 food categories, shown in Table 1.

For each unique product label, the information of product name, manufacturer, package size and list of ingredients was obtained. Additionally, information about the sort of bread was obtained for sandwiches. Food composition data and nutritional values were recorded for each unique product label and normalised per 100 g of food (energy, fat, saturated fatty acids, sugars, salt, protein, dietary fibre, amount of fruits and vegetables provided in the ingredient list). Additionally, the amount of trans fatty acids (TFAs) in specific food was estimated using the database compiled for the previously reported assessment of TFAs in the Slovenian food supply [34].

### 2.2. Nutrient Profiling

Two nutritional quality indicators were used to assess the nutritional quality of the foods: We used a Traffic-light Food Labelling (TFL) system, which is also used in Slovenia (supporting the interpretation of nutrition declaration with a smartphone application [35] (Table 2)). This system targets key nutrients of concern (fat, saturated fatty acids, sugar and salt), which are coloured in traffic light colours, depending on the level of a specific nutrient (i.e., high level of a nutrient is presented in red, and low level in green).

The second model for nutrient profiling was the Food Standards Australia New Zealand Nutrient Profiling Scoring Criterion (FSANZ), which was originally developed to determine the eligibility of foods to be labelled with health claims [36,37]. This is a scoring model; foods receive positive and negative points for energy, key nutrients and constituents, and there are different (sum) score thresholds for three types of products (beverages, foods, and fats and cheese with high calcium content). For this study, foods that did not pass the FSANZ criterion were considered as less healthy. This nutrient profiling model was used recently for an assessment of the nutritional quality of foods available in the Slovenian food supply [38].

### 2.3. Data Processing and Analyses

Data were collected and processed in Microsoft Excel 16.0. (Microsoft Corporation, Redmond, WA, USA) and R 3.6.0 (R Foundation, Vienna, Austria). For each food category, we calculated average content (±standard deviation; SD) of energy (kJ), sugar, fat, salt and dietary fibre. We also calculated the per-category proportion of less healthy foods according to FSANZ criteria. Results of profiling with the Food traffic light are presented separately for vending machines in different health and social care institutions. We calculated the proportion of foods, assigned with green/red/amber colour for each of the four evaluated nutrients (fat, saturated fatty acids, sugar and salt). Although we sampled all available foods in the vending machines, the results are provided as absolute numbers, without providing confidence intervals.

## 3. Results

Out of the 188 institutions surveyed, 71.3% (*n* = 134) had at least one vending machine at the visited location. Vending machines were not present in 7.7% of hospitals (2 out of 26), 12.5% health centres (8 out of 64) and 44.9% nursing homes (44 out of 98). From the total of 5625 products present in the vending machines, 45.85% (*n* = 2579) products were foods (i.e., 48.3% in hospitals, 47.2% in health centres and 42.6% in nursing homes) and were further investigated in this study. Other products in vending machines were beverages, which were included in a separate study [39]. Out of 2579 food products, 29.4% (*n* = 758) were found in hospitals, 38.7% (*n* = 998) in health centers and 31.9% (*n* = 823) in nursing homes vending machines. After exclusion of 45 products present in vending machines (consisting of 5 unique food items) not labelled with nutrition declaration, the final study sample contained 2534 foods presented in vending machines, of which there were 267 unique food products.

Nutritional composition of foods displayed in vending machines in health and social care institutions is presented in Table 3. Most frequently displayed types of foods in the vending machines were chocolate and sweets (30.5%), biscuits (28.5%) and crisps and snacks (11.4%), followed by nuts and seeds (8.4%), cereal bars (8.2%), pre-prepared salads and sandwiches (5.9%), chewing gum (2.8%) yoghurt products (2.1%), fruits (1.4%), ice cream and edible ices (0.5%) and milk (0.3%). The highest energy and fat contents were observed in chocolate and sweets (2084 ± 322 kJ/100 g and 26.5 ± 9.0 g/100 g, respectively), highest sugar content in fruit (65.5 ± 16.0 g/100 g), and highest salt content in crisps and snacks (2.4 ± 0.9 g/100 g). The amount of trans fatty acids (TFAs) was estimated using food labelling information (on the use of partially hydrogenated oils, which are a source of industrial TFAs) and a food composition database, compiled in another study, where TFAs were measured using laboratory analyses [28]. Out of 267 unique foods in our dataset, nine were manufactured using partially hydrogenated oils, and are expected to contain above 2 g TFAs/100 g fat. In two of these, high TFA levels were confirmed in a previous study [34]—one in the category biscuits (found in 14 vending machines), and one in the category of crisps and snacks (found in 12 vending machines). It should be noted that since 2019 the amount of TFAs in foods is limited by regulation according to which the maximum permitted TFA level in foodstuffs is 2 g per 100 g of total fat content in the foodstuff [20,40].

When applying the FSANZ nutritional profiling model, we determined that the majority of available foods can be assigned to be less healthy (Table 3). In fact, 88.5% of all displayed food items in vending machines can be considered as of lower nutritional quality or as less healthy products. Foods within the categories ice cream and edible ices, chocolate and sweets, biscuits, crisps and snacks, and cereal bars were almost exclusively (>95%) identified as less healthy. A very high proportion of less healthy foods was also observed in yoghurt products and pre-prepared salads and sandwiches. Out of all assessed yoghurt products, 85.5% can be considered as less healthy, given that 9 out of 10 yoghurts are flavoured yoghurt products with high sugar content (red TFL indicator). 88.2% of ready to eat salads and sandwiches were assessed to be less healthy as the majority of sandwiches are stuffed with meat (prosciutto, ham, salami, sausages) (69%) and mostly made with white flour bread (73%). Sandwiches filled with fried cheese or chicken (16%), tuna sandwiches (11%) and also vegetarian sandwiches (4%) should be a healthier alternative. Still, because of their high salt content (average 1.6 g per 100 g) meaning red TFL indicator, only 11.8% of such sandwiches are evaluated as healthy by FSANZ nutritional profiling model. However, the majority of nuts and seeds (63.9%), fruit (59.5%) were assigned as healthy, despite containing high average energy, fat or sugar content per 100 g (red TFL indicator), as these are the consequences of their favourable fatty acid composition in nuts and seeds, and fruits/vegetables/nuts content. All assessed kinds of milk and chewing gums are healthy products, according to FSANZ nutritional profiling model.

**Figure 1:**
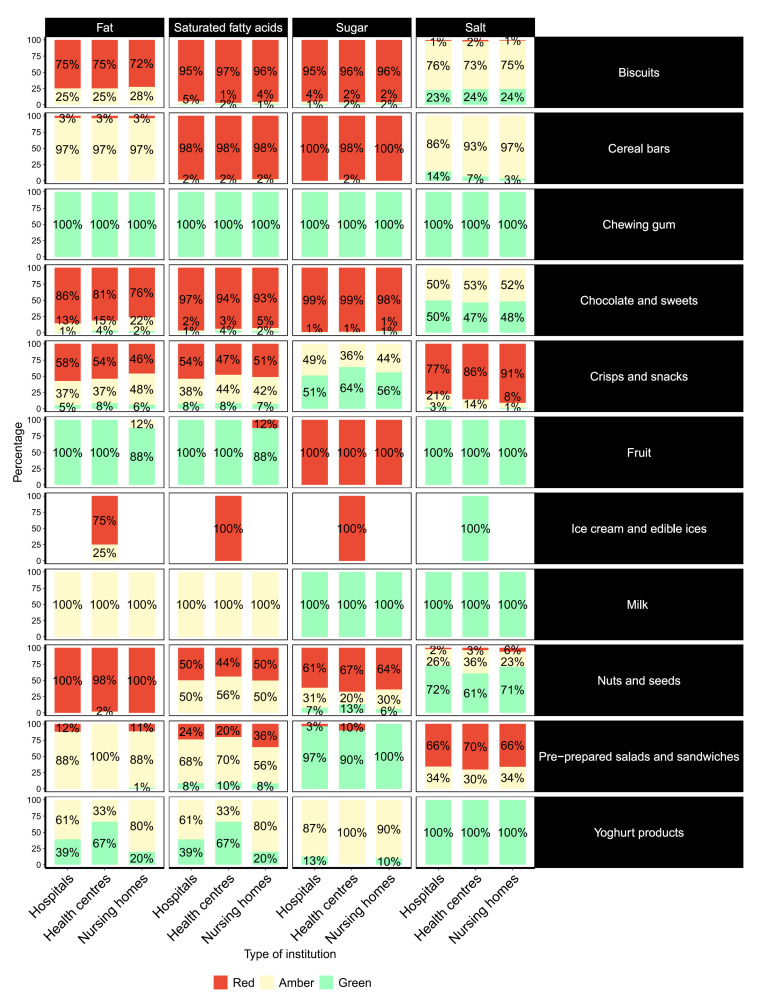
Percentage (%) of displayed foods (per food category) in the vending machines in health and social care institutions assessed as green/amber/red (food traffic light profiling).

Using TFL indicators for fat, saturated fats, sugar and salt content, we can observe that the majority of the categories (7 out of 13 categories) were foods high in the nutrients of concern. Exceptions are foods from categories of fruit, milk, yoghurt products and chewing gums. The majority of biscuits, cereal bars, chocolates, sweets, and ice creams fall in the red category of the TFL system regarding fat and/or saturated fat content. Although the majority of nuts and seed are also categorised red for fat content, those products are better categorised (amber) in terms of saturated fat content. The majority of these same categories also fall into the red labelling regarding sugar content. Fruits represent a special group, with red labelling regarding sugar content, but this at the expense of naturally occurring sugar. However, between 60% and up to 91% of crisps, snacks, pre-prepared salads and sandwiches are labelled red regarding salt content (Figure 1).

## 4. Discussion

Vending machines are very convenient to supply food and drinks in places where people are having limited (or limited time to) access food stores. This study investigated the food supply in vending machines in health and social care institutions in Slovenia. We have shown that food products represent almost half of the products in vending machines; therefore future activities should be directed towards an increased share of healthy foods in vending machines. The ratio between healthy and unhealthy food products is currently just the opposite, as sweet products dominate (ca. 70%), while nuts and seeds (8.4%), yoghurts (2.1%), fruits (1.4%) and milk (0.3%) are either present in very small proportions or are not available at all. These results are in line with other international studies in other countries, where most common snacks vended were also salty snacks and sweets [1,3,5,7,41,42,43]. Confectionary and snack foods are typical ultra-processed foods, which should be limited in one’s diet due to their negative impact on human health [44,45] and not promoted, as it seems when looking at offers at the examined vending machines.

The share of cereal bars offered, which could be healthier alternatives, is currently at a satisfactory level (8.2%) regarding the official guidelines in preparation in Slovenia, but products offered in this category should not contain greasy and sweet toppings, which is the situation at the moment. Comparing the nutritional profile of cereals and cereal products available in Slovenian food supply markets, vending machines offer products of lower nutritional quality with 99.5% evaluated as less healthy, according to the FSANZ nutrient profile model. In comparison, in the Slovenian food supply (in food markets) only 23% of such products were identified as less healthy [38]. This result indicates that there is much room for improvement, which can be improved by properly selecting and marketing healthier alternatives in vending machines. The situation is similar to the supply of yoghurt products, where the proportion of healthier offers should increase. Above all, natural yoghurt products should lead above flavoured milk fermented products. Regarding sandwiches, the choice of bread portion would greatly contribute to improving the composition of sandwiches. This was already pointed out by students in a survey at Slovenian faculties. Students clearly stated that they miss the offer of sandwiches in general, as they are not available at all faculties. They suggested sandwiches, not necessarily made of white bread and stuffed with greasy spreads, but contain more fresh salads and, last but not least, a larger offer of vegetarian and vegan sandwiches [46]. High average sugar, energy and fat content in the majority of ultra-proceed foods (which are dominating in vending machines) contribute to worldwide obesity and overweight [16] and are recognised as individual risk factors for obesity [17].

While high free sugar intake has been shown to be a public health issue globally, and in Slovenia [47], it should be mentioned that vending machines investigated in our study also contained a very high proportion of foods with high sugar content. Additionally, vending machines also supply many sugar-containing drinks (35 preprint). Additional factors of concern with regards to diet risks are that foods in vending machines are often also high in salt and saturated fats. A population study of excreted sodium in 24 h-urine a decade ago showed that the adult population of Slovenia exceeds the recommended daily amount of salt intake by more than 130%, where recommendations for intake are no more than 5g of salt for adults and less than 2 g of salt for children [48,49]. It is estimated that the majority of salt intake in developed countries is related to the consumption of processed foods [50]. As we have not made significant progress in reducing salt content in processed foods [51], we expect that salt intake in the population has not changed much since then. By consuming food containing more than 1.5 g of salt per 100 g of the product (red TFL label), both children and adults can easily exceed recommended salt intake. This represents an important risk factor for the development of chronic non-communicable diseases [49]. The World Health Organization (WHO), together with member states, have set a goal of reducing salt intake by 30% globally by 2025, preventing 2.5 million deaths annually [52]. To achieve this goal, pricing and other strategies targeting the food supply (i.e., reducing the price or increasing the availability of healthier choices) are proposed [26,27,28,53,54,55,56].

The intake of saturated fat (SF) in the Slovenian population was estimated recently in a national dietary study named “SI.Menu 2017/2018” [57]; it is estimated that SF account to about 11% of total energy intake [58]. Our study showed that vending machines also offer a wide variety of foods high in saturated fats, particularly in categories biscuits, cookies, cereal bars, chocolate and snacks, ice cream and edible ices, ready to eat salads and sandwiches.

Although the results of this present study comply with the finding of other research on vending machine offers [1,3,7,41,42,59], we were also able to observe some positive exceptions and examples of good practice. For example, 5 institutions offered plain yoghurts in vending machines, but only 2 unique products were found. Nuts in small portions were found in vending machines at more than half the institutions (103 institutions, offering 18 unique products), but the share of these products in relation to all foods listed were only 8.38%. Among fresh fruits, 17 institutions were offering apples, and 5 institutions were offering smoothies (3 unique products with no added sugar). It is necessary to take into account that providing healthier options with shorter shelf-life like salads, fresh fruit, and yoghurt requires technical adaptations of vending machines, which are already available on the market. Refrigerated food vending may offer an opportunity to have fresh, healthy foods in vending, rather than having to rely on the limited range of pre-packaged items [60]. We strongly believe that with 2579 unique food items offered in vending machines at the moment, there are many opportunities to improve the vending machine offers and to upgrade vending machines into a tool for the promotion of healthy eating. A promising strategy for achieving this goal are altering the availability of vending machine products by removing the unhealthy products from the current offers and add new healthy products. While shops and cafes are open during the daytime in hospitals, at night, vending machines are often the only option available from which staff, visitors, or patients can purchase food [5,41,61]. Also, while waiting in line at health care institution, one does not have time to purchase in the shop or café. Therefore, the majority of hospitals (24 out of 26) and health centres (56 out of 64) in Slovenia have at least one vending machine placed inside the institution. The situation in nursing homes, with a different type of population and activities, is somewhat different, in that only 54 out of 98 nursing homes had at least one vending machine.

Promoting healthier choices has proven to be an effective strategy for improving consumer choices in vending machines [27,30,56], especially in hospitals. Vending machines offering healthy snacks are feasible in hospital settings [62]. By altering the availability of foods and drinks by increasing the range of healthier food/drink options and/or decreasing the range of less healthy options, the overall sales of healthier items can increase [61] with no loss of overall sales volume [26,63]. Although the loss of profit is the main counter-argument of vending companies, increasing the number of healthier options does not necessarily negatively impact the overall revenue [56,64,65]. To support these findings, our future activities within a frame of the National Program on Nutrition and Physical Activity for Health, will be focused on comparing the sales of products from the classic and the pilot vending machines with healthier choices. It should be noted that official guidelines are being prepared and will be proposed to the Ministry of Health, providing recommendations related to the tendering conditions for vending machines in health and social care institutions, which will ensure wider availability of healthier foods and beverages.

A key strength of this study is that we were able to include almost all vending machines in health and social care institutions in Slovenia (except for only one location, where permission was withheld). This resulted in a very large and representative dataset, which enabled an in-depth assessment of the food supply. While this database is representative only for the Slovenian food supply, we need to mention that being part of the European Union market resulted in the fact that this dataset also contains all major international food brands. A limitation of this study is that foods were not analysed as we used the information about food composition provided by food manufacturers on food labels.

## 5. Conclusions

Vending machines are considered an important environmental factor that contributes to the availability of nutrient-poor and energy-rich food products. In contrast, healthier products are rarely available or not available at all. An effective strategy for improving consumer choices in vending machines, especially in hospitals, has proven to be promoting healthier choices by altering the availability of vending machine products. Briefly, we suggest that vending machines are mostly filled with healthier choices like non-sugared beverages, fresh fruits, vegetables and dairy products. The snacks offered should represent a healthy choice with low sugar, salt and fat content. Sandwiches should be filled with meat and fish with visible structure, cheese or vegetarian in small portions with less salt and more fibres. Also, the portion sizes of all offered products should be limited to suit a smaller snack.

Based on the above findings, we propose that regulatory guidelines should be included in the tender conditions for vending machines in health and social care institutions, to ensure healthy dietary choices provided to patients, staff and visitors.

## Figures and Tables

**Table 1:** Foods categorisation.

(Sub) Category	Description/Examples
Biscuits	biscuits, cookies, croissant, wafer
Cereal Bars	cereal tiles
Chewing Gum	chewing gum
Chocolate and Sweets	candy, chocolate, chocolate/ chocolate snacks, fruit tiles
Crisps and Snacks	chips, crackers, flips, salted sticks/salty pretzels
Fruit	dried fruits, fresh fruits
Ice Cream and Edible Ices	ice cream
Milk	milk
Nuts and Seeds	nuts
Pre-prepared Salads and Sandwiches	fried cheese/chicken, prosciutto or ham, salami and sausages, tuna, vegetarian
Yoghurt Products	fruit, natural

**Table 2:** Criteria for traffic light labelling of foods in Slovenia.

Quality Indicator	Green	Amber	Red
	(g/100 g)		
Fat	<3	3–20	>20
Saturated Fatty Acids	<1	1–5	>5
Sugar	<5	5–15	>15
Salt	<0.3	0.3–1.5	>1.5

**Table 3:** Nutritional assessment of foods displayed in vending machines in health and social care institutions (*n* = 2534) according to food categories.

Food Category							
	Total Share in Relation to All Foods Listed (%)	Energy (kJ) *	Sugar (g) *	Fat (g) *	Salt *	Dietary Fiber *	% Less Healthy Foods by FSANZ ***
Chocolate and Sweets	30.5% (*n* = 787)	2083.8 (SD = 321.9)	46.5 (SD = 11.1)	26.5 (SD = 9.0)	0.3 (SD = 0.2)	3.6 (SD = 3.4)	97.5
Biscuits	28.5% (*n* = 736)	1928.7 (SD = 344.5)	32.4 (SD = 13.0)	23.7 (SD = 6.9)	0.4 (SD = 0.3)	2.5 (SD = 1.4)	98.9
Crisps and Snacks	11.4% (*n* = 294)	1957.3 (SD = 282.5)	3.7 (SD = 2.4)	20.9 (SD = 10.6)	2.4 (SD = 0.9)	2.8 (SD = 0.7)	99.0
Nuts and Seeds	8.4% (*n* = 216)	2184.4 (SD = 188.6)	21.4 (SD = 13.7)	36.8 (SD = 7.8)	0.3 (SD = 0.5)	4.9 (SD = 1.3)	36.1
Cereal Bars	8.2% (*n* = 211)	1245.4 (SD = 712.7)	32.4 (SD = 3.5)	14.7 (SD = 3.1)	0.4 (SD = 0.1)	4.1 (SD = 2.7)	99.5
Pre-prepared Salads and Sandwiches	5.9% (*n* = 152)	1140.8 (SD = 176.4)	2.5 (SD = 3.6)	11.9 (SD = 4.3)	1.6 (SD = 0.4)	2.4 (SD = 4.2)	88.2
Chewing Gum	2.8% (*n* = 71)	627.2 (SD = 17.7)	0 (SD = 0)	0 (SD = 0)	0 (SD = 0)	0 (SD = 0)	0.0
Yoghurt Products	2.1% (*n* = 55)	326.6 (SD = 36.5)	10.2 (SD = 2.5)	2.6 (SD = 1.1)	0.1 (SD < 0.1)	/	85.5
Fruit	1.4% (*n* = 37)	1337.1 (SD = 162.5)	65.5 (SD = 16.0)	1.6 (SD = 3.4)	0 (SD < 0.1)	/	40.5
Ice Cream and Edible Ices	0.5% (*n* = 12)	1433.4 (SD = 232.2)	27 (SD = 5.0)	22.7 (SD = 5.2)	0.1 (SD < 0.1)	/	100.0
Milk **	0.3% (*n* = 8)	266 (SD = 0)	4.7 (SD = 0)	3.5 (SD = 0)	0.1 (SD = 0)	/	0.0

* average content per 100 g; SD = standard deviation. ** Nutritional data values for milk are presented per 100 mL *** Proportion of the displayed foods that did not pass Food Standards Australia New Zealand Nutrient Profiling Scoring Criterion (FSANZ).
